# Pollen Overload: Seasonal Allergies in a Changing Climate

**DOI:** 10.1289/ehp.124-A70

**Published:** 2016-04-01

**Authors:** Charles W. Schmidt

**Affiliations:** Charles W. Schmidt, MS, an award-winning science writer from Portland, ME, writes for *Scientific American*, *Science*, various *Nature* publications, and many other magazines, research journals, and websites.

Watery red eyes, runny nose, sneezing, coughing—these familiar symptoms mean spring is in the air. Millions of people suffer from seasonal allergies triggered by airborne pollen—not just in spring but in summer and fall, too—and now evidence suggests their numbers will rise in a changing climate. The evidence so far is preliminary, but it points to a confluence of factors that favor longer growing seasons for the noxious weeds and other plants that trigger seasonal allergies and asthma attacks. Carbon dioxide (CO_2_), in addition to being the principal global warming gas, can also be thought of as plant food—it’s the source of carbon needed to make sugars during photosynthesis.^1^ When exposed to warmer temperatures and higher levels of CO_2_, plants grow more vigorously and produce more pollen than they otherwise would.^1^^,^^2^

**Figure:**
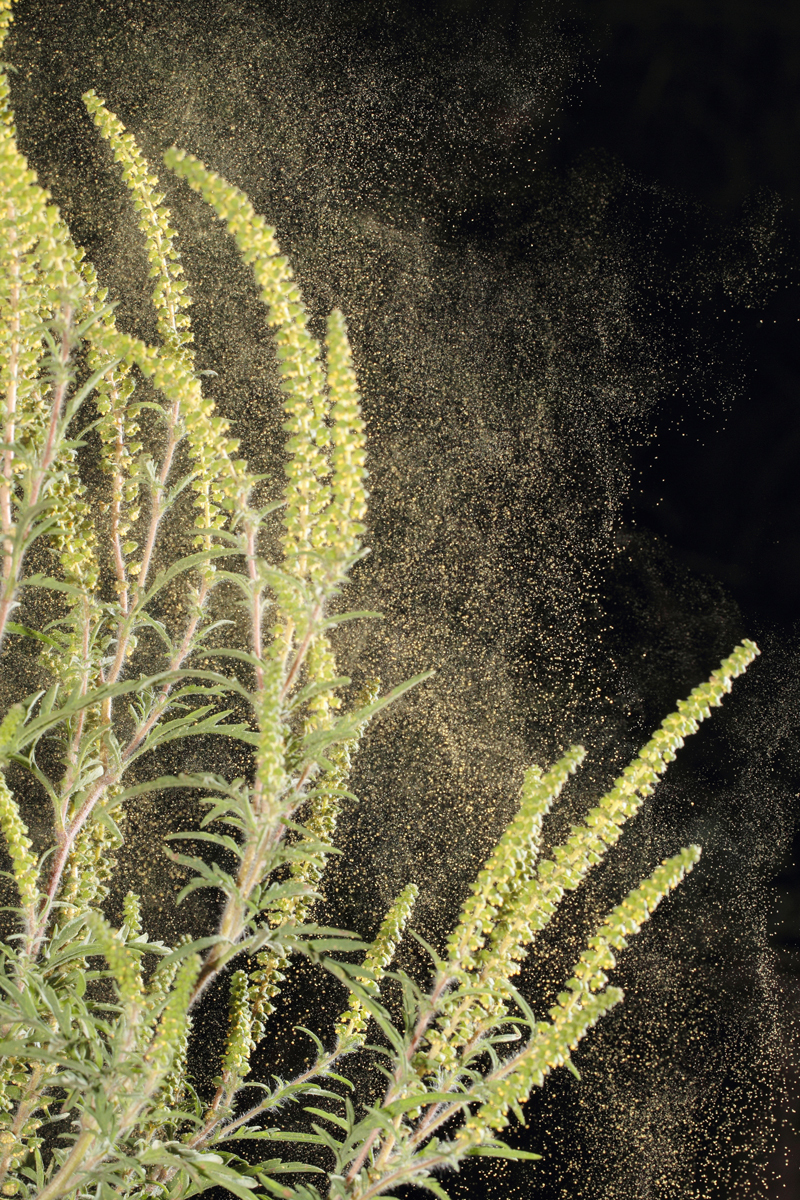
Several intertwined factors influence how a plant grows and how much pollen it produces. Projected rises in two of these—regional temperatures and atmospheric CO_2_ levels—could mean an increase in the number of people affected by seasonal allergies. © Joe Petersburger/Getty

Physicians who treat allergic airway diseases are already reporting an uptick in symptoms that they attribute to climate change.^3^ In a statement published last year, the World Allergy Organization, comprising 97 medical societies from around the world, opined that climate change will affect the start, duration, and intensity of the pollen season and exacerbate the synergistic effects of pollutants and respiratory infections on asthma.^4^

“We’re seeing increases in both the number of people with allergies and what they’re allergic to,” says Leonard Bielory, a professor and allergy specialist at the Rutgers University Center of Environmental Prediction and attending physician at Robert Wood Johnson University Hospital. “Should warming continue,” he says, “then more people will be exposed to seasonal allergens with subsequent effects on public health.”

## Allergies on the Rise

Seasonal allergies and asthma impose significant health burdens, with an estimated 10–30% of the global population afflicted by allergic rhinitis (or hay fever) and 300 million people worldwide affected by asthma.^5^ Trend data suggest that the prevalence of asthma, including forms of the disease triggered by pollen, mold, and other allergenic substances, is on the rise. Childhood asthma rates in the United States, for instance, doubled from 1980 to 1995 before slowing to a more gradual (albeit ongoing) increase.^6^ Kate Weinberger, a postdoctoral associate at Brown University, says trends in seasonal allergy prevalence are more difficult to track because symptoms in most cases don’t trigger emergency room visits or other types of medical care.

**Figure:**
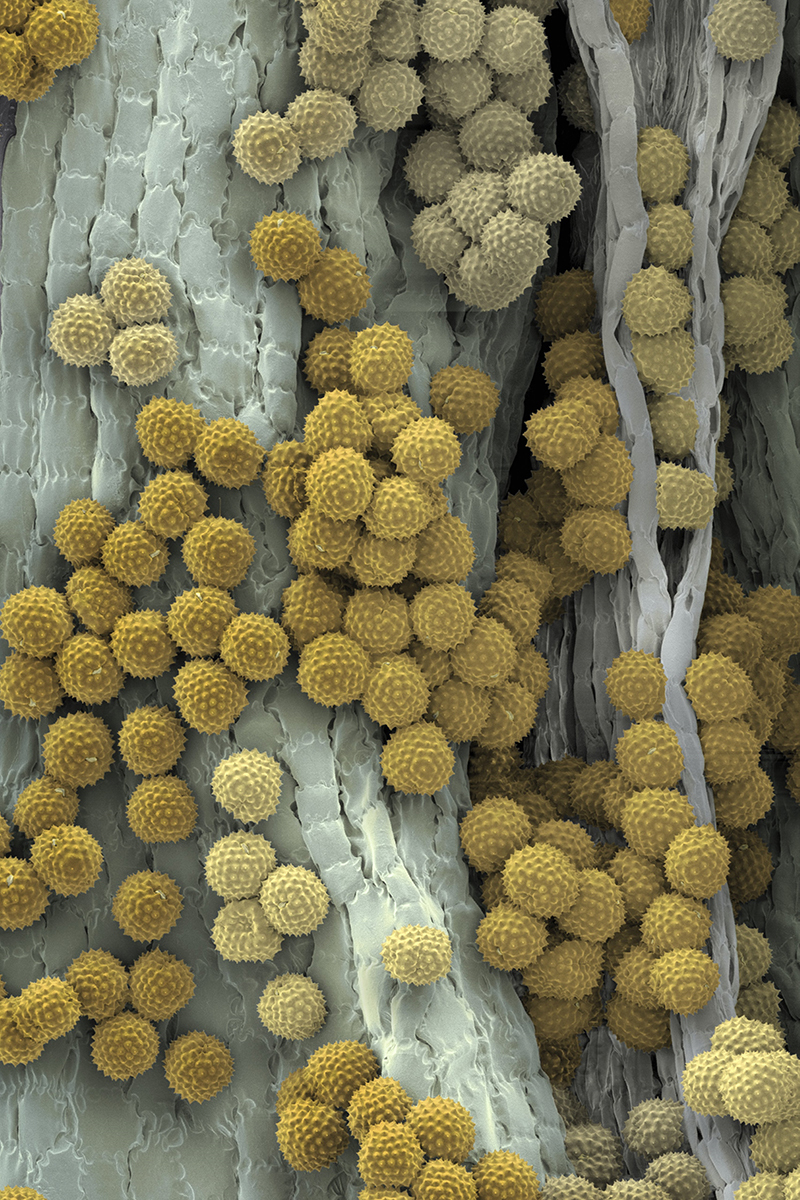
Pollen (gold spheres) is produced by the stamens (gray), which are the male reproductive organ of flowering plants. Pollen grains are covered in proteins that assist in reproduction but also trigger allergic reactions in sensitized people. © Martin Oeggerli/Science Source

There is evidence suggesting that hay fever prevalence is rising in many parts of the world, particularly in urban areas, although some of the most recently published studies date back to the late 1990s.^7^^,^^8^^,^^9^ A newer report from France’s Rhône-Alpes Center of Epidemiology and Health Prevention shows that hay fever prevalence rose from 8% of the local population in 2004 to 12% in 2015.^10^ Michel Thiboudon, director of the French National Aerobiological Monitoring Network, attributes the rising prevalence to increased exposures to highly allergenic ragweed. Climate change has been projected to accelerate ragweed’s spread throughout the European continent.^11^

Bielory says it’s likely that other environmental factors, such as changing diets and better hygiene, contribute to the prevalence of asthma and hay fever by limiting early exposure to allergens and altering the immune system’s normal development. However, much remains unknown about the relationship between aeroallergens and exacerbation of asthma, especially less severe attacks that aren’t reflected in hospital visit data.^12^

Seasonal allergies in North America generally begin in spring, when trees begin to flower and disperse their allergenic pollen into the air—they include, among others, oak and birch in the South and Northeast and mountain cedar in the West. Late spring and early summer bring the emergence of various allergenic grasses and weeds, such as mugwort and nettle, which introduces another round of symptoms. The ragweed season comes last, starting in late summer and persisting until the plants die with the first frost.^13^ A resurgence in grass pollen also occurs in early fall, Bielory says.

Pollen grains contain the male gametes (sperm cells) of the flowering plant; they are covered in proteins that female gametes of the same species will recognize. It’s those same coating proteins that trigger allergic reactions in sensitized people, with the degree of sensitization varying among individuals. According to Lewis Ziska, a research plant physiologist with the U.S. Department of Agriculture (USDA), the intensity of an allergic reaction depends on three interrelated factors: how much pollen a given species emits into the air, the duration of exposure, and the allergenicity of the pollen. In ragweed these factors coalesce in a perfect storm of allergic misery. “What’s unique about ragweed is that it produces so much pollen—roughly a billion grains per plant,” Ziska says. “And the Amb a 1 protein [contained in the ragweed pollen coat] is also highly reactive with the immune system.”

## Regional Differences

Ziska conducted studies in the 1990s to explore potential links between pollen production, rising CO_2_ levels, and warming temperatures. He grew ragweed in chambers containing up to 600 ppm ambient CO_2_. That’s the atmospheric concentration that the Intergovernmental Panel on Climate Change predicts by the year 2050, assuming no changes in current emissions.^14^ (At present, the atmospheric concentration level is just over 400 ppm.^15^) Ziska found that the size of the experimental ragweed plants and their pollen output increased in tandem with rising CO_2._^16^

**Figure:**
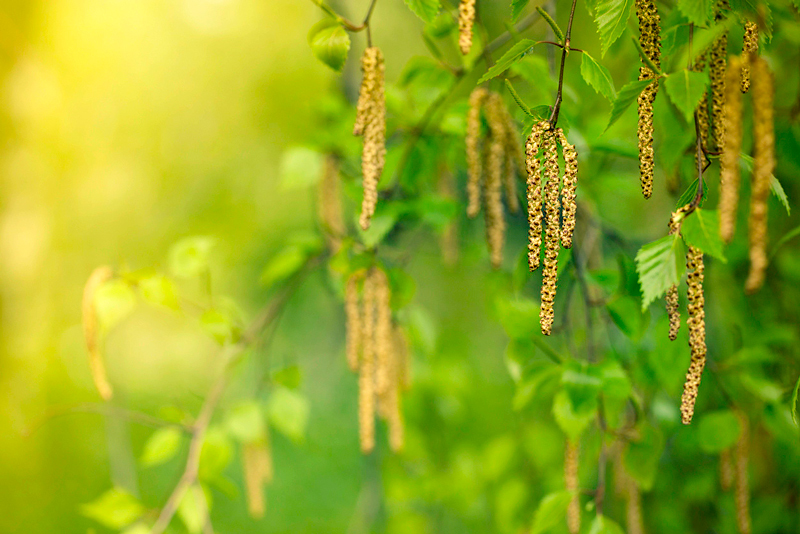
Seasonal allergies in North America begin in the spring, when trees such as birch (shown), oak, and mountain cedar begin to bloom. The inconspicuous flowers of plants like these are designed not to attract pollinators but to release their pollen into the air, where it is carried by the wind. © Marcus Lindstrom/Getty

**Figure:**
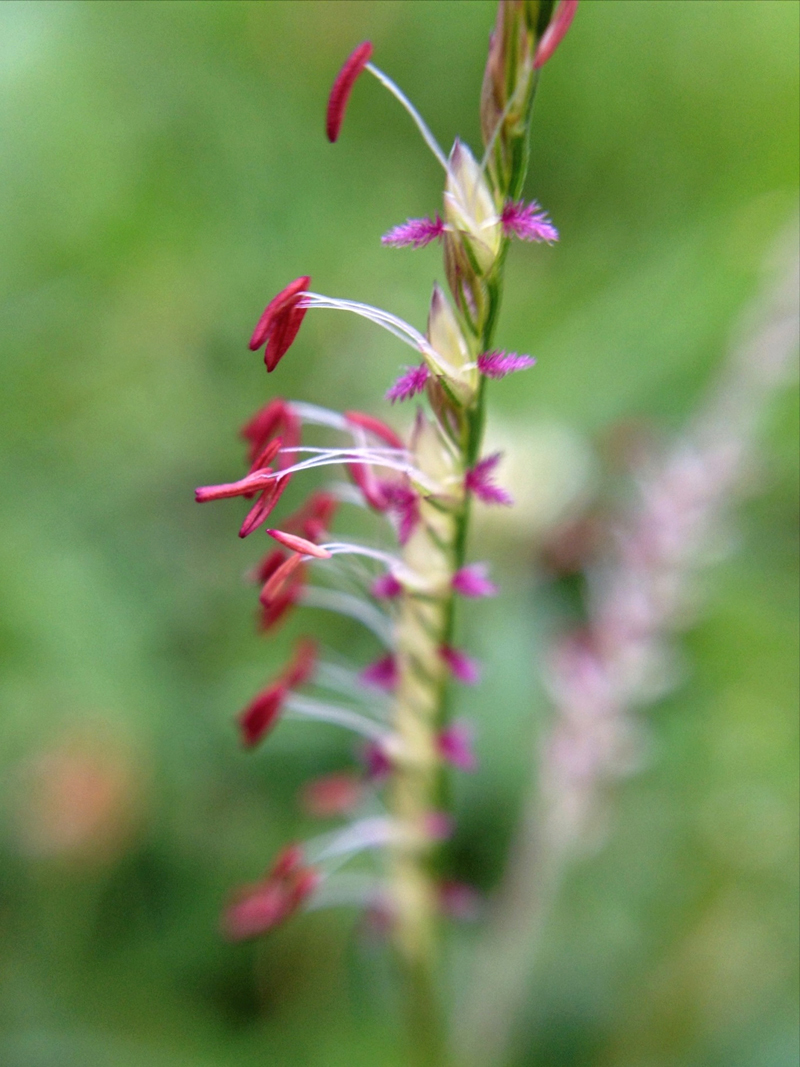
Certain grasses and weeds (such as Bermuda grass, shown here magnified) trigger further allergies into the summer. Flowers that are barely visible to the naked eye can pack a major allergenic punch. © Susan M. Booker

Ziska then modeled future climate conditions using a novel surrogate: He and his colleagues compared how ragweed grew in urban and rural locations. Their rationale was that cities are heat sinks (because they’re paved in dark surfaces that absorb and later re-radiate solar heat) as well as sources of CO_2_ (from traffic and industrial emissions). Ziska’s team planted ragweed in urban Baltimore, where measured CO_2_ levels were 30% higher and temperatures 3.5°F hotter on average than they were outside the city. Their findings showed that urban ragweed plants grew faster, flowered earlier, and produced more pollen than those grown outside the city.^17^

Climate change–related warming is anticipated to increase as one moves up in latitude.^18^ To assess the effect of warming temperatures on the length of ragweed’s flowering season, Ziska’s team, including Bielory, studied measures of airborne pollen collected from 10 sampling stations extending from east Texas to Saskatoon, 2,200 kilometers to the north. The results, though not unexpected, were remarkable: Between 1995 and 2009, they found the pollen seasons lengthened by 13–27 days, with greater increases the farther north they looked.^13^

During a more recent study published in 2014, Bielory and colleagues reached a similar conclusion. This team studied pollen measures taken from 50 sampling stations across the contiguous United States between 1994 and 2010. They reported that pollen seasons for allergenic species were lengthening more in the north than in the south, and that total counts of daily airborne pollen were getting larger. As Ziska’s research also showed, the lengths of the southern pollen seasons were either unchanged or had actually shortened with time.^2^

That finding goes to the heart of the geographic complexities underlying climate change and its influence on biological systems. Ziska explains that CO_2_ and atmospheric water vapor exert competing influences on warming trends—water vapor suppresses warming in wetter, rainier southern latitudes, in part by boosting cloud cover, while CO_2_ accelerates warming in dryer regions farther from the equator.

The implications of these phenomena are consistent with the health data. Jonathan Silverberg, an assistant professor at Northwestern University Feinberg School of Medicine, and his colleagues studied rates of childhood hay fever in relation to pollen counts and weather conditions throughout the United States, and found they were lowest in wetter areas with higher humidity levels.^19^

Meanwhile, in Europe ragweed has dramatically expanded its range since it was first introduced to the continent in the 1800s,^20^ and scientists anticipate its spread will accelerate further with climate change. Modeling by the French Climate and Environment Sciences Laboratory predicts a four-fold jump in levels of airborne ragweed pollen by 2050, with the biggest increases occurring in northern and eastern parts of Europe.^11^ Apart from ongoing seed dispersal, the models estimate higher CO_2_ levels and warmer temperatures will help lengthen ragweed’s pollen season.

A study published in 2014 showed that pollen seasons have already become longer in western Poland.^21^ The authors focused on allergenic species other than ragweed—namely, nettle, sorrel, broad-leaf dock, and various grasses. According to their sampling results, species-specific pollen seasons lengthened by 2 to nearly 4 days between 1996 and 2011, a trend the authors attributed mainly to warmer summer temperatures and later pollen season end dates.

## Accessing Pollen Data

European pollen databases are more accessible and widespread than those in the United States, says Richard Flagan, a professor of environmental science and engineering at the California Institute of Technology. He says that’s chiefly because European national weather agencies take responsibility for sampling and organizing the information in ways that scientists can use for research.

By contrast, pollen sampling in the United States is performed by a constellation of agencies and allergy clinics. Currently 84 of these sampling stations submit their data to a volunteer organization called the National Allergy Bureau™ (NAB), which is organized by the American Academy of Allergy, Asthma & Immunology (AAAAI).^22^ Bielory says the AAAAI provides quality control in the form of training and certification for contributors on how to sample airborne pollen.

**Figure:**
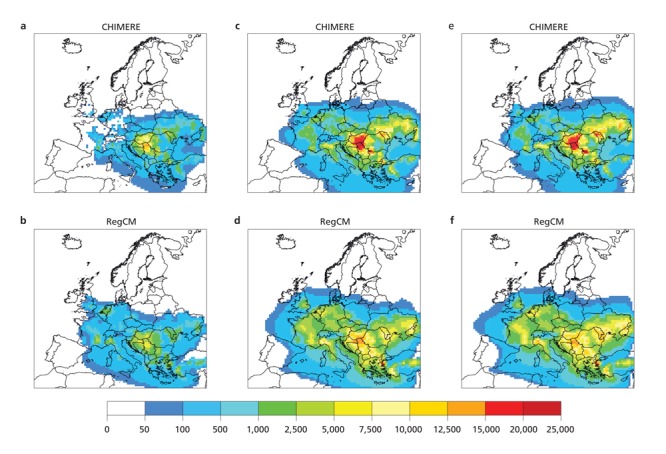
Using two different climate models, researchers modeled historical levels of ragweed pollen in Europe (frames a/b), then projected increases by 2050 (frames c/d and e/f). Some areas saw a projected fourfold jump. Source: Hamaoui-Laguel et al. (2015)^11^

The NAB provides daily pollen counts to local media outlets, but it won’t release any data for research without the consent of the sampling stations that collected it. To access those data, scientists have to submit formal requests describing their research plans.^23^ The NAB passes approved requests to the appropriate member stations, which have 30 days to respond.

Flagan describes his efforts to access NAB data as “an exercise in frustration” that was frequently met with unanswered phone calls and e-mails. “Moreover, the way these stations collect data isn’t compatible with science,” he says. “We have at best a semi-qualitative historical record supplied by people who do not focus on the statistics of the measurement—that record has some scientific value, but you have to look at it with a big grain of salt. In reality, the pollen database in the United States is abysmal.”

The USDA’s Ziska says the NAB has become more cooperative and responsive to the needs of outside researchers. But he adds that since NAB sampling stations use different tools and methodologies to collect pollen, rather than one uniform system, their data can be difficult to aggregate and compare.

Bielory, who contributes to the NAB, agrees on the need for a national monitoring system that collects, stores, analyzes, and shares pollen data for the purpose of advancing science and health policy issues. The Council of State and Territorial Epidemiologists, a professional association for public health epidemiologists, has proposed such a system in a draft white paper that it plans to finalize at its June 2016 annual conference.

## Lab Results Hint at Possibilities

Even as researchers grapple with limited field data, they continue to produce compelling results in climate-controlled chambers that predict future effects on allergenic species. In her research at the University of Massachusetts Amherst, Kristina Stinson, an assistant professor of environmental conservation, grows ragweed in greenhouses containing CO_2_ at levels ranging from 360 ppm—just under the current ambient concentration—to 720 ppm. Stinson says higher CO_2_ levels could force evolutionary changes in ragweed. A study she published in 2011 showed that genotypes that are suppressed at current CO_2_ levels devoted more resources to reproduction as CO_2_ levels rose.^24^ In other words, she says, more genotypes overall were flowering. Stinson says that while she didn’t measure pollen output directly, “we do note that more vigorous flowering and higher pollen production are usually correlated.”

**Figure:**
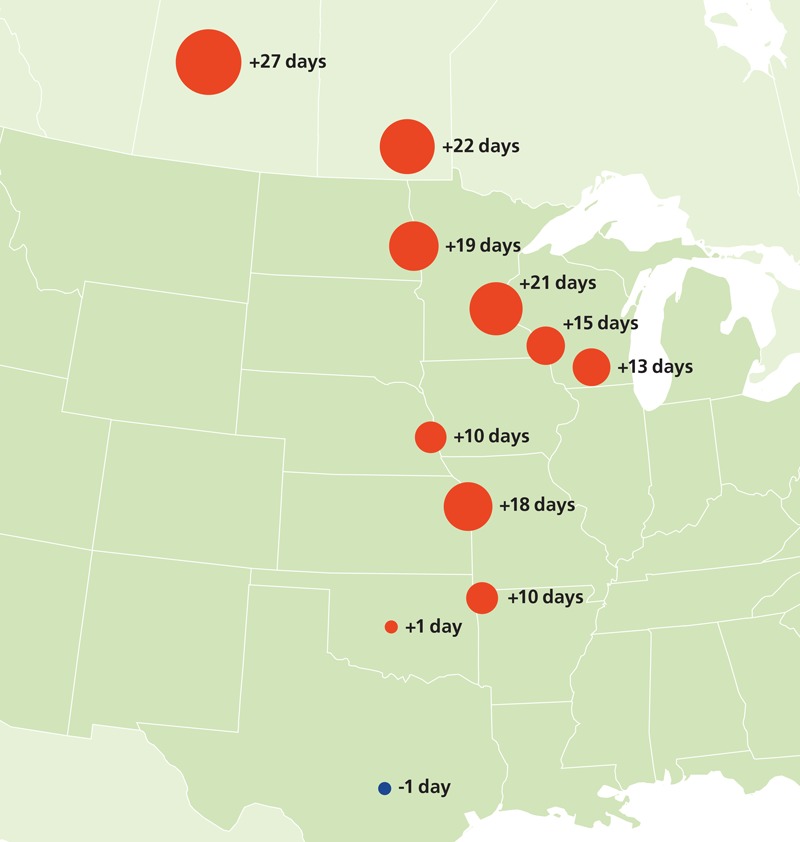
Warming is expected to increase with increasing distance from the equator. One multiyear study in North America found that pollen seasons lengthened incrementally with increasing northern latitude—by up to 4 weeks—while decreasing slightly in the southernmost monitoring location. Source: U.S. EPA, based on data from Ziska et al. (2011)^13^,^11^

Her colleague Jennifer Albertine, a postdoctoral researcher at the University of Massachusetts Amherst, generated comparable results with timothy grass, a widespread perennial in North America and Europe and a major cause of early summer allergies. Albertine studied the effects of CO_2_ at both 400 and 800 ppm. She found that timothy grass exposed to 800 ppm CO_2_ produced roughly twice as much pollen as the lower-exposed grass.^25^

Albertine also tested the effects of boosting ground-level ozone, which ordinarily slows plant growth by inducing oxidative damage. Coupled climate/tropospheric chemistry modeling indicates ozone levels could rise significantly by the end of the century as emissions of precursor pollutants also continue rising.^26^ Albertine’s study didn’t reveal any growth-limiting effect of ozone on grasses raised in elevated CO_2._ But she did find that the grasses responded to higher ozone levels by making less of their allergenic protein (Phl p 5). However, any reduction in the plant’s allergenic protein content, Albertine predicted, would be offset by a corresponding increase in pollen production, for a net boost in allergenic threat.^25^ (Similarly, Ziska’s research showed that when raised in greenhouses containing up to 600 ppm CO_2,_ ragweed plants produced 60–80% more of their allergenic protein, Amb a 1.^27^)

Stinson acknowledges that, although greenhouses allow for a controlled assessment of how atmospheric conditions affect allergenic plants, they don’t replicate the real world, where other pollutants, humidity, rainfall, and additional soil nutrients—especially nitrogen—also influence plant growth and pollination patterns. With funding from the U.S. Environmental Protection Agency, she’s now collaborating with David Foster, director of the 3,750-acre Harvard Forest, on a project to map ragweed hot spots in New England. Their field studies so far, which have been submitted for publication, show that ragweed plants from urban and rural areas differ in the extent and timing of flower production and in their responses to CO_2._

Among other research questions, Stinson hopes to explore spatial patterns in how people experience the effects of climate change on pollen production. “We may find that urban populations from a particular demographic might be disproportionately affected by how climate change affects allergenicity,” she says.

## Connecting the Dots on Health

Stinson says that connecting climate-induced trends in allergenicity with public health impacts could be challenging. It will require that scientists have better access to pollen data than they currently do in addition to health outcomes data that might be correlated with rising pollen exposure levels.

**Figure:**
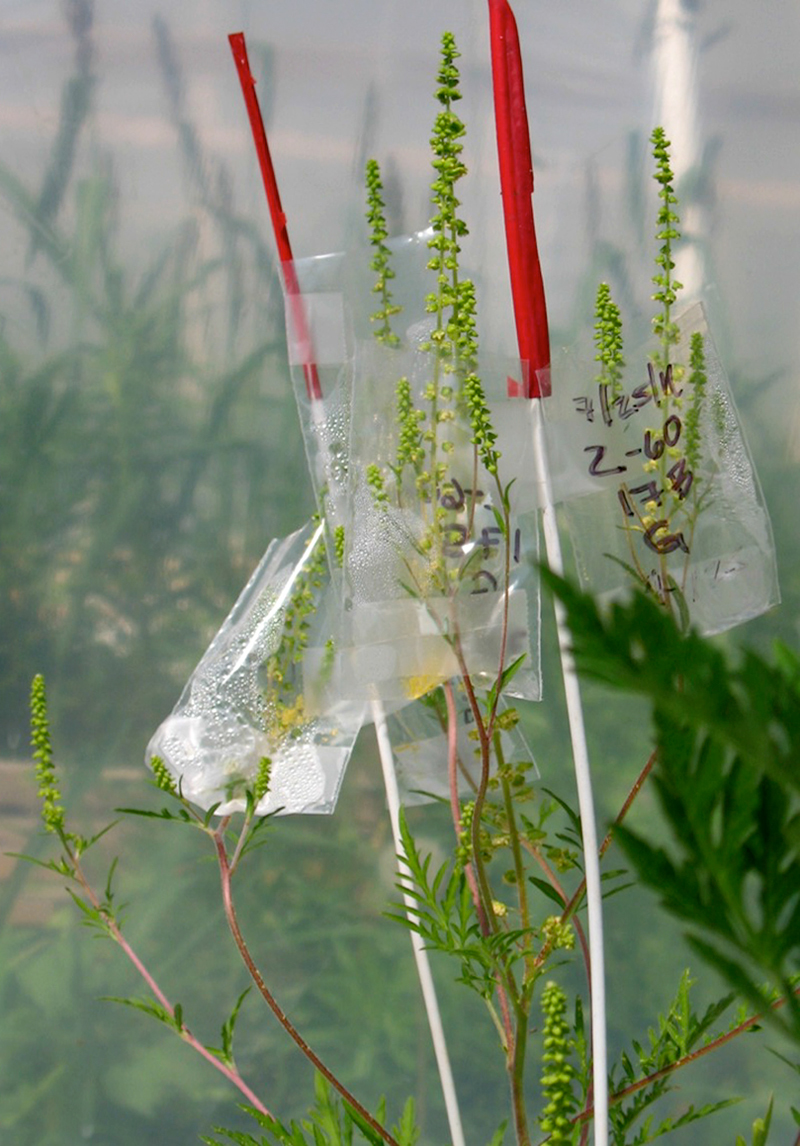
Growing ragweed in greenhouses enables researchers to study potential effects of higher ambient CO_2_ levels. But it can’t replicate the complex interplay of real-world conditions that determine pollen production. © David McLain/Aurora Photos

**Figure:**
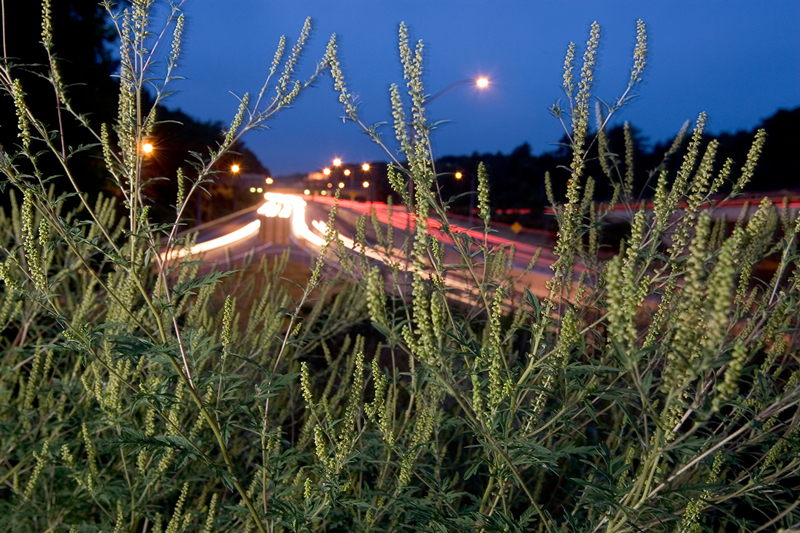
Findings from studies of ragweed in urban versus rural settings suggest that some city dwellers might be disproportionately affected by climate change with respect to seasonal allergies. © Kristina Stinson

Weinberger, of Brown University, has studied the relationship between daily spring pollen counts and health outcomes in New York City. Results published last year showed that mid-spring peaks in tree pollen were associated with over-the-counter allergy medication sales and emergency room visits for asthma attacks, especially among children.^28^ By contrast, unpublished research she’s conducted showed no similar relationship between allergy drug sales and peak exposures to ragweed pollen in the fall. Weinberger says that’s possibly because allergy medication purchased in the spring might last for months; in the absence of sales data, researchers wouldn’t be able to detect a relationship to symptoms.

Despite the data gaps that remain, many healthcare professionals believe the trend is real, as evidenced by surveys of physicians who treat seasonal allergies. One survey involved members of the American Thoracic Society, including pulmonologists, critical care clinicians, pediatricians, and other specialists. Over half the participants queried in the survey reported increases in allergic symptoms among their own patients that the doctors believed were related to climate change.^3^ A survey of AAAAI members, currently in press, reached a similar conclusion: In this case, specialists were asked “[How] do you think your patients are being affected by climate change or might be affected in the next 10–20 years?” Nearly two-thirds reported seeing “increased care for allergic sensitization and symptoms of exposure to plants or mold.”^29^

Mona Sarfaty, director of the Program on Climate and Health at George Mason University, led both those surveys. She says that to her surprise, neither study detected regional difference in physician responses. “Instead, greater allergy symptoms seemed to be showing up across the country,” she says, with only the symptoms themselves varying by location. “So a doctor in Michigan who ordinarily sees relief from mold allergies with the arrival of cold weather might see them persisting later into the year,” she explains, “while a doctor in Southern California might be reporting grass allergies all year round.” Sarfaty says that doctors who claimed not to believe in climate change were less likely to report these trends.

Kim Knowlton, a senior scientist with the Natural Resources Defense Council, who also holds a faculty post at the Columbia University Mailman School of Public Health, acknowledges the need for more research. “What we have to do is tease out the chain of events starting with higher temperatures and CO_2_ levels, to effects on allergenicity, to human health symptoms,” she says. “The studies so far are compelling, but we need more comprehensive studies at larger scales.” For the tens of millions who have allergies and asthma, this is more than an inconvenience, she says—“Those illnesses can keep you out of school and work, and for some they are absolutely life-threatening. So these are really substantial health concerns.”
